# Use of Atomic Force Microscopy to Study the Multi-Modular Interaction of Bacterial Adhesins to Mucins

**DOI:** 10.3390/ijms17111854

**Published:** 2016-11-08

**Authors:** A. Patrick Gunning, Devon Kavanaugh, Elizabeth Thursby, Sabrina Etzold, Donald A. MacKenzie, Nathalie Juge

**Affiliations:** 1 The Gut Health and Food Safety Institute Strategic Programme, Institute of Food Research, Norwich Research Park, Norwich NR4 7UA, UK; Devon.Kavanaugh@ifr.ac.uk (D.K.); Elizabeth.Thursby@ifr.ac.uk (E.T.); sabrinaetzold1@gmail.com (S.E.); d.mackenzie91@btinternet.com (D.A.M.); 2 Division of Neonatology and Division of Gastroenterology, Hepatology and Nutrition, Department of Pediatrics, School of Medicine, University of California San Diego, 9500 Gilman Drive, San Diego, CA 92093-0715, USA

**Keywords:** atomic force microscopy, single molecule force spectroscopy, intestinal mucin, mucus binding protein, bacterial adhesins, gut microbiota, *Lactobacillus reuteri*

## Abstract

The mucus layer covering the gastrointestinal (GI) epithelium is critical in selecting and maintaining homeostatic interactions with our gut bacteria. However, the molecular details of these interactions are not well understood. Here, we provide mechanistic insights into the adhesion properties of the canonical mucus-binding protein (MUB), a large multi-repeat cell–surface adhesin found in *Lactobacillus* inhabiting the GI tract. We used atomic force microscopy to unravel the mechanism driving MUB-mediated adhesion to mucins. Using single-molecule force spectroscopy we showed that MUB displayed remarkable adhesive properties favouring a nanospring-like adhesion model between MUB and mucin mediated by unfolding of the multiple repeats constituting the adhesin. We obtained direct evidence for MUB self-interaction; MUB–MUB followed a similar binding pattern, confirming that MUB modular structure mediated such mechanism. This was in marked contrast with the mucin adhesion behaviour presented by Galectin-3 (Gal-3), a mammalian lectin characterised by a single carbohydrate binding domain (CRD). The binding mechanisms reported here perfectly match the particular structural organization of MUB, which maximizes interactions with the mucin glycan receptors through its long and linear multi-repeat structure, potentiating the retention of bacteria within the outer mucus layer.

## 1. Introduction

The human gastrointestinal (GI) tract is the main microbial niche in the body and is estimated to contain up to 10^14^ microbial cells constituting the gut microbiota [1]. The gut microbiota plays a key role in metabolic and immunological pathways, and host protection against pathogens. An alteration of the gut microbiota composition or “dysbiosis” has been linked to a number of intestinal disorders such as inflammatory bowel disease (IBD) [2].

The large intestine is lined by a bi-layer of mucus, with the outer layer providing a habitat to bacteria, whereas the inner layer maintains them at a safe distance from the epithelial surface [3]. The *O*-glycan structures present in mucin are diverse and complex, consisting predominantly of core 1–4 mucin-type *O*-glycans containing α- and β- linked *N*-acetyl-galactosamine, galactose, and *N*-acetyl-glucosamine. These core structures are further elongated and frequently modified by fucose and sialic acid sugar residues via α1,2/3/4 and α2,3/6 linkages, respectively [4]. The terminal mucin *O*-glycans have been proposed to serve as attachment sites and metabolic substrates to the gut commensal bacteria which have adapted to the mucosal environment [5,6].

Reflecting the structural diversity of mucin glycans and their prime location, gut bacteria have evolved a range of cell–surface adhesins allowing their interaction with mucus including S-layer proteins [7,8], teichoic and lipoteichoic acids [9,10], pili [11,12,13,14], fimbria [15], moonlighting proteins [16,17,18,19,20], or lectins [21,22]. However, detailed information of the nature of the glycan receptors is often lacking [5,23].

One of the best studied examples of mucus adhesins in commensal/probiotic bacteria is the canonical mucus-binding protein, MUB, produced by *Lactobacillus reuteri* ATCC 53608. MUB is a large, modular, cell surface protein (~350 kDa) made up of 14 Mub repeats of ~20 kDa, divided in two types (Mub1 and Mub2) based on sequence identity and an N-terminal domain of unknown function [24,25]. Small angle X-ray scattering (SAXS) and atomic force microscopy (AFM) demonstrated a “beads on a string” arrangement of the Mub repeats, generating ~174 nm long protein fibrils [26]. The binding of the full-length MUB to mucus appears to be mediated via multiple interactions involving terminal sialylated mucin glycans, as shown by the net reduction in MUB adhesion to (1) mucin-secreting epithelial cells grown in the presence of an inhibitor of sialylation; or to (2) mammalian intestinal tissue after chemical desialylation [26]. However, direct measurements of MUB binding to mucin glycans are lacking. In addition MUB has been implicated in the ability of bacterial strains to auto-aggregate as demonstrated by flow-cytometry using *L. reuteri* ATCC 53608 wild-type and MUB-deficient mutant strain, 1063N [25].

Methodologies to screen for bacterial adhesion to mucins have previously employed thin layer chromatography overlay [26], enzyme-linked immunosorbent assay [15], micro-titre plate assays [8,25,27], surface plasmon resonance [16,28,29,30,31], fluorescence spectroscopy [20], mucin microarrays [32], flow cytometry [33], and cell-based assays [26,34,35,36]. However, due to the complexity and diversity of mucin glycosylation, these methods typically provide qualitative binding data indicating only presence or absence of interaction. In recent years, AFM has become a method of choice to decipher the complex interactions occurring between mucins and bacteria/bacterial adhesins at the nanoscale [37]. The force measurement carried out by AFM is a form of spectroscopy because it collects a combination of force, distance and time which can provide more details of molecular interactions [38] than the traditional methodologies which are used to discover molecular interactions. AFM was recently used to investigate the pili-mediated binding of gut bacterial cells from *Lactobacillus rhamnosus* GG and *Lactococcus lactis* to mucins [39,40,41] or to explore the spatial distribution of mucin glycans [42].

In the current study, AFM has been used to demonstrate the interaction of a large modular cell–surface adhesin (MUB from *L. reuteri* ATCC 53608) to intestinal mucins, providing novel insights into the nature of the interaction to mucin glycans.

## 2. Results

In order to assess the binding of MUB to mucins, a novel purification protocol was established to obtain pure amounts of native MUB from *L. reuteri* ATCC 53608 bacterial cells. Briefly, stepwise ammonium sulphate precipitation was included to remove a large proportion of the contaminating substances, including proteins, lipids, and glycolipids, at 20% and precipitate MUB at 60%. Three phase partitioning, employing 20% ammonium sulphate to re-suspend the precipitate followed by pure *t*-butanol, resulted in a MUB-enriched interface, with nearly all of the residual contaminating glycolipid found in the upper *t*-butanol layer. The use of 0.5% CHAPS in the size exclusion eluent resulted in a clean elution peak in the void volume for MUB, the purity of which was confirmed by SDS-PAGE (Figure S1).

**Figure 1:**
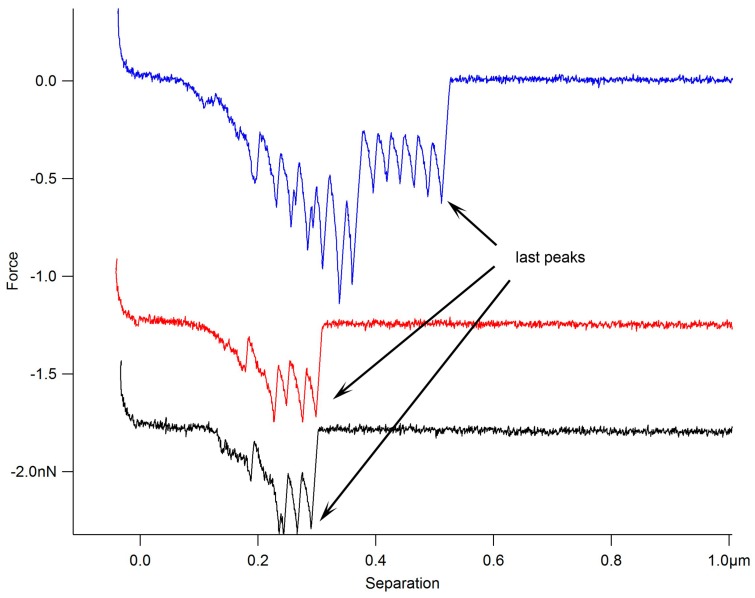
Example of retraction force-distance curves of mucus-binding protein (MUB) unfolding. Typical force-distance curves were obtained when probing the interaction between MUB and pPGM in phosphate buffered saline (PBS) (**blue curve**) α2,6-sialyllactose (6’SL) (100 mM, **red curve**) and a mixture of 3’SL (50 mM) and 6’SL (50 mM) (**black curve**). The red and black curves are offset for clarity. The symmetrical nature of the negative peaks suggests that MUB is unfolding. The “last peaks” are the “rupture” events of MUB’s interaction with pPGM. The effect of the free sugars on the interaction between MUB and pPGM suggests that the binding is mediated by protein–carbohydrate interactions with terminal mucin glycans.

**Figure 2:**
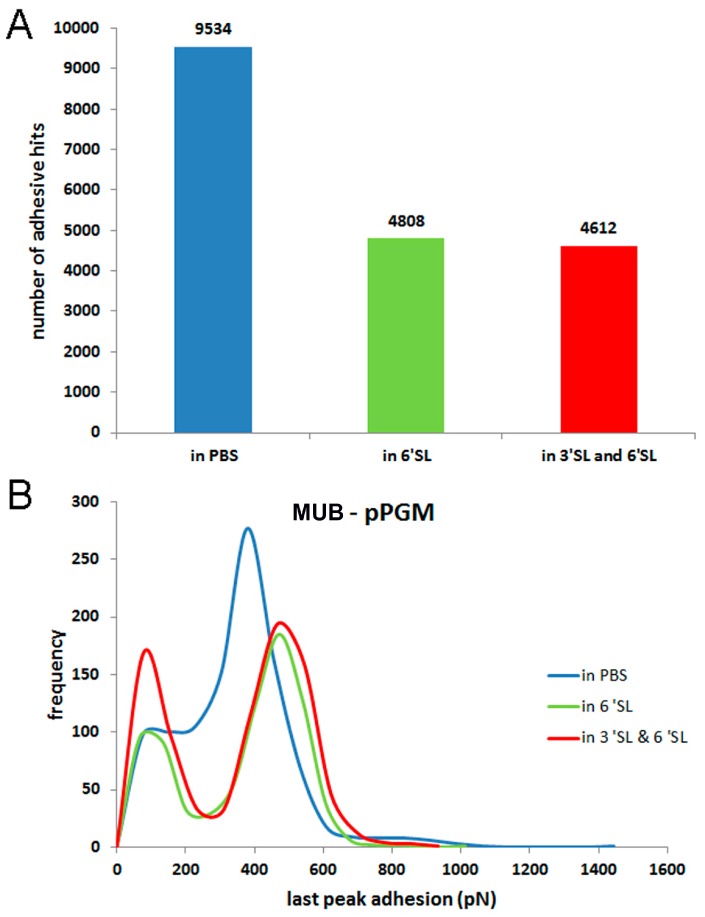
Atomic force microscopy (AFM) force spectroscopy of MUB–pPGM in the absence or presence of 3’SL and 6’SL (**A**) Quantification of the number of all negative peaks in the retraction force curves (defined as adhesive hits); (**B**) Histograms of MUB–pPGM “rupture” adhesion forces showing the effect of adding competing sugars in a free solution state on the interaction of MUB to mucin glycan receptors.

Force spectroscopy was used to assess MUB binding to purified porcine gastric mucin (pPGM) by immobilising mucin on the glass slides and MUB on the AFM functionalised tips, taking advantage of specific covalent attachment chemistry [43]. The force mapping was repeated in the same area of the pPGM coated glass slide to enable precise comparison of the interaction changes which occur after adding free molecules to the solution. Figure 1 shows example force-distance retraction curves of MUB–mucin binding obtained in phosphate buffered saline (PBS) and also in the presence of α2,3-sialyllactose (3’SL) and α2,6-sialyllactose (6’SL). The addition of the sialylated sugars reduced the number of multiple adhesion peaks. Furthermore, the symmetrical multiple negative peaks seen in the retract curves revealed that the MUB protein becomes unfolded as it is pulled apart from the mucin due to the strength of the binding, with the “last peaks” reflecting the “rupture” events. The initial binding positions can be random as the two molecules meet each other upon approach in the force distance runs. It is thus expected that not every single domain will be unfolded in the force curves upon retraction. In addition, the reduced unfolding events observed after the addition of the sialylated sugars provide direct evidence of the competitive effects of the free sugars. Figure 2A illustrates the quantification of the number of all the multiple adhesive events which occur in each of the force-distance measurements. The quantification of the last peaks (i.e., “rupture” adhesive events) shows the significant number of “rupture”adhesive events that occurred at relatively high strengths (modal value of 380 pN in PBS) (Figure 2B). Addition of free 3’SL and 6’SL did not cause a reduction in the highest adhesion magnitude (high modal values of 467 pN), but reduced the frequency of the “rupture” peaks from 277 to 194 and 185, respectively, resulting in a clear bimodal distribution (lower modal value of 78 pN).

**Figure 3:**
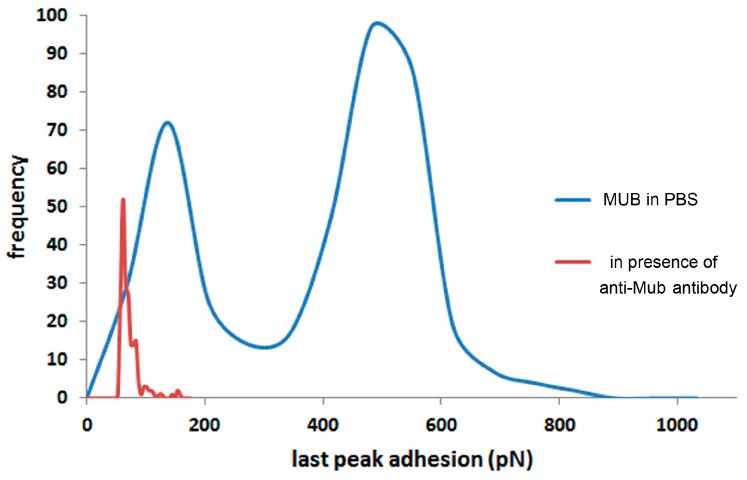
Effect of anti-Mub antibody on the interaction between MUB and pPGM. The histogram data represent the quantification of the MUB–pPGM “rupture” adhesion in the retraction force curves measured between the MUB tip and the mucin coated slide in PBS or in the presence of anti-Mub antibody in the solution.

Since there are multiple binding sites on both the MUB and mucin molecules, it is likely that addition of higher concentration of sugar combinations will be necessary to cause total inhibition. Such inhibition of binding was shown here by addition of anti-Mub antibodies to the solution (Figure 3). The modal values of adhesion dropped from 482 to 61 pN.

**Figure 4:**
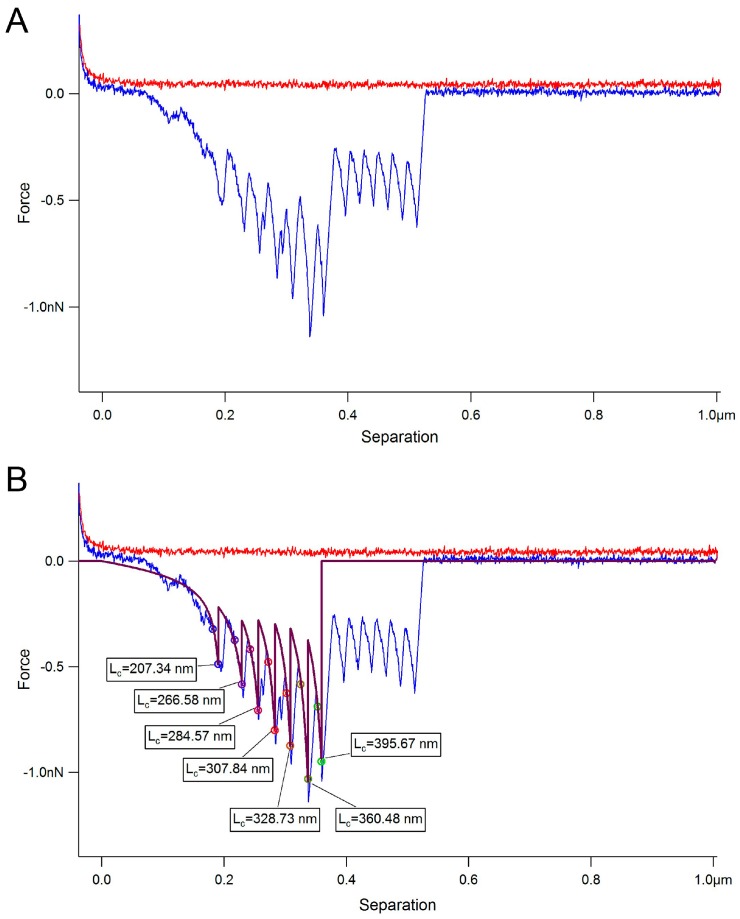

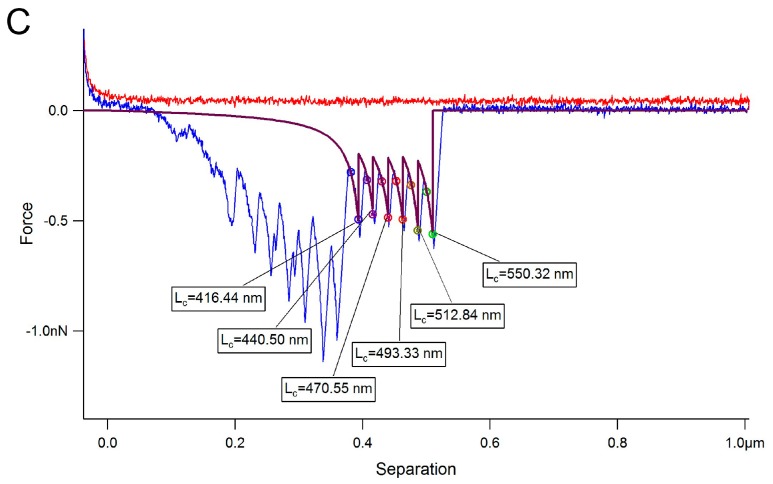
Modelling unfolding of MUB. (**A**) Example force-distance curve of MUB interacting with pPGM; (**B**) Measurement of the contour length (*L_c_*) in the first set of unfolded domains; and (**C**) Measurement of the contour length (*L_c_*) in the second set of unfolded domains. Fitting the worm-like-chain (WLC) mathematical model to the negative peaks in the retraction force distance curves (**blue**) enables quantification of the contour and persistence lengths of the unfolding of each domain.

**Figure 5:**
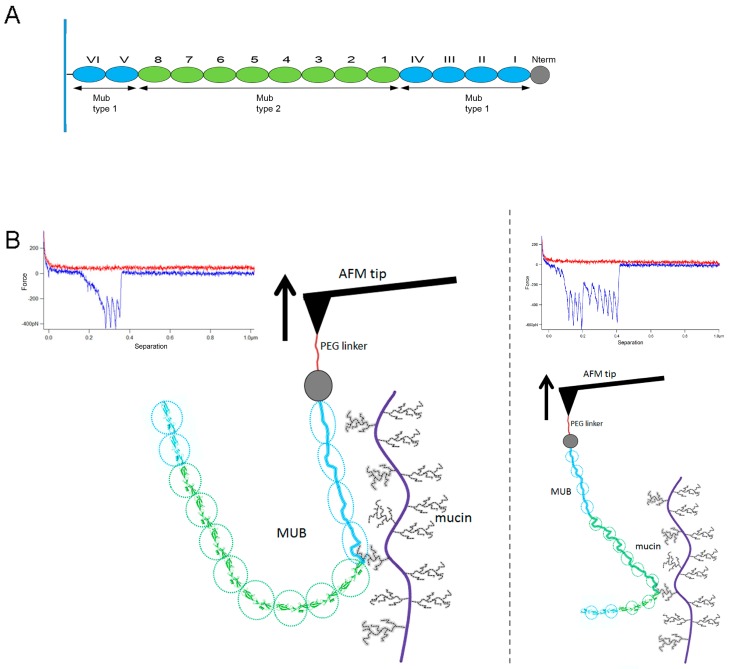
Diagram of the proposed binding scheme. (**A**) Schematic representation of the modular repeat region of MUB from *L. reuteri* ATCC 53608 (**grey**: N-terminal domain; blue: type 1 Mub repeat domains; green: type 2 Mub repeat domains) and (**B**) Schematic diagram representation of the negative peaks in the force curve. **Left** panel: unfolding of four type 1 Mub repeat domains (**blue**); **Right** panel: unfolding of four type 1 Mub repeat domains (**blue**) and six type 2 Mub repeat domains (**green**).

Adhesion force peaks are well-described by the worm-like-chain (WLC) model. The stretching behaviour of semi-flexible polymers is described by two statistical models, the Kratky-Porod WLC model [44,45] and the freely-jointed chain (FJC) [46,47] models. Formula describes the extension, *z*, of a worm-like chain with contour length *L_c_* and persistence length, *l_p_*, in response to a stretching force, *F*, as:

(1) $$F(z)=\frac{k_{B}T}{l_{P}}\left[\frac{1}{4}{\left(1-\frac{z}{L_{c}}\right)}^{-2}+\frac{z}{L_{c}}-\frac{1}{4}\right]$$

where, *k_B_*, is Boltzmann’s constant and *T*, absolute temperature. Previous research on protein unfolding uses the WLC model [48]. Figure 4 shows the methodology used to measure the unfolding distances of MUB multiple repeat domains. Figure 4A shows an example of force-distance curve. The negative peaks in the retraction curves are fitted to the WLC model to determine the contour length (*L_c_*). The distance of separation between the contour lengths is 25.6 ± 5.8 nm in the first section (the longer peaks in Figure 4B) and 26.6 ± 6.8 nm in the second section (the shorter peaks in Figure 4C) which is approximately double the size of each Mub repeat based on the X-ray crystal structure of type 1 and type 2 Mub repeats [26]. These data are consistent with the unfolding of Mub repeat domains, although it is not possible to assign with certainty which type repeats are being unfolded. Please note that the binding of MUB to mucin is stochastic and thus it is not expected that all repeat domains will be unfolded upon retraction (see also Figure 5 for other examples of force–distance curves).

Based on these data, we propose a schematic explanation of how the number and size of the peaks may reveal which repeat domains constituting the MUB protein (Figure 5A) are bound to the mucin. In Figure 5B, the exemplar force-distance curve in the left panel shows four peaks which may correspond to the unfolding of the first four type 1 Mub repeat domains upon retraction, as depicted in the schematic (Figure 5B). The diagram in the right panel is based upon an exemplar force-distance curve with ten negative peaks, which may correspond to the sequential unfolding of the first four type 1 Mub repeat domains and six type 2 Mub repeat domains (Figure 5B).

**Figure 6:**
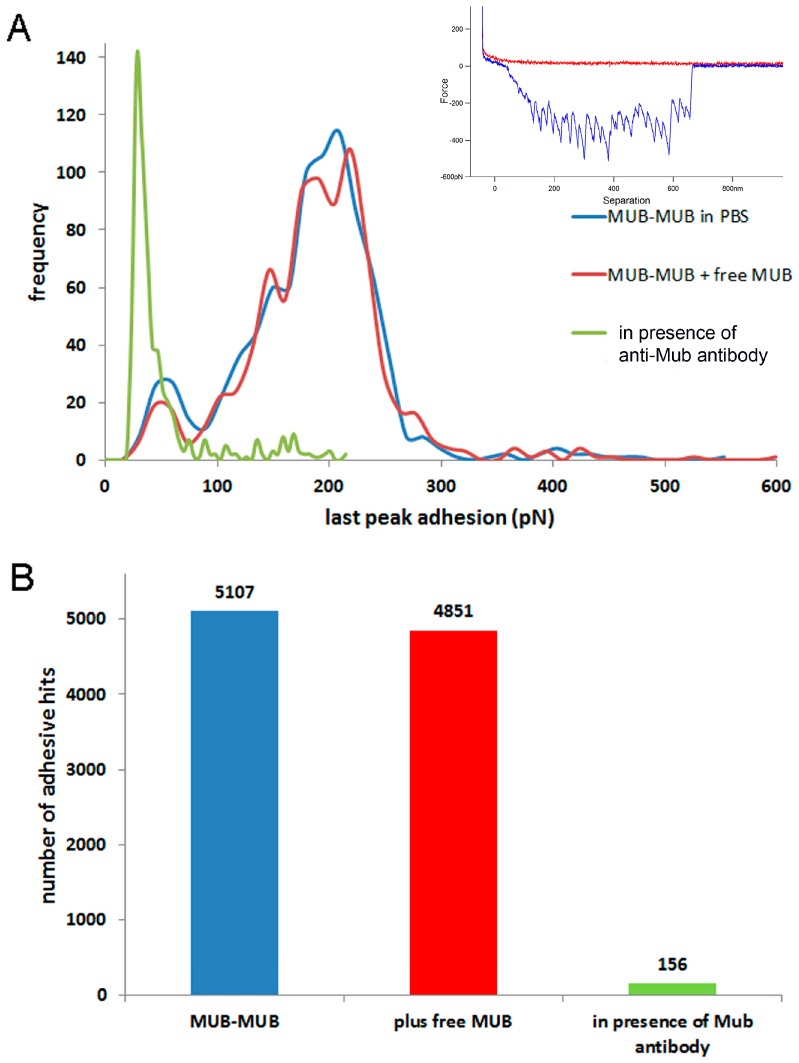
AFM force spectroscopy of MUB–MUB in absence or presence of free MUB or anti-Mub antibody. (**A**) Histograms of the “rupture” adhesion forces. Inset: example force curve and (**B**) Quantification of the numbers of unfolding negative peaks (defined as adhesive hits). These are calculated from the retraction force distance curves showing the effects of the addition of free MUB and anti-Mub antibodies to the solution.

The unfolding binding mechanism presented by MUB was further confirmed in MUB self-interaction experiments where force volume was mapped between a MUB functionalised AFM tip across a region of a covalently attached MUB slide (Figure 6). The inset example of force curves (Figure 6A) shows a higher number of negative peaks than the number of repeat domains in a single MUB molecule. This may be explained by the fact that when two MUB molecules interact, both sets of their respective repeat domains can unfold until they are pulled apart. The modal value of the adhesion magnitude was 209 pN (Figure 6A) and a significant number of negative peaks (defined as adhesive hits) due to unfolding events were observed (Figure 6B). Addition of anti-Mub antibody to the liquid cell caused a dramatic reduction in the adhesion magnitude (Figure 6A modal value 28 pN) below the noise level of the force curves whereas addition of free MUB did not impact on the adhesion values. This is in line with the ability of an anti-Mub antibody to block interaction sites along the protein length, whereas free MUB will self-interact with the MUB present on the tip or glass slide. In addition, quantification of the number of negative peaks (Figure 6B) showed a consistent reduction of MUB self-interactions in the presence of anti-Mub antibody. As generally expected for antibody-protein interactions, the association rate of anti-Mub antibody remaining attached to MUB was significantly longer (13 s) than observed for protein–carbohydrate interactions (Figure S2) [49,50].

**Figure 7:**
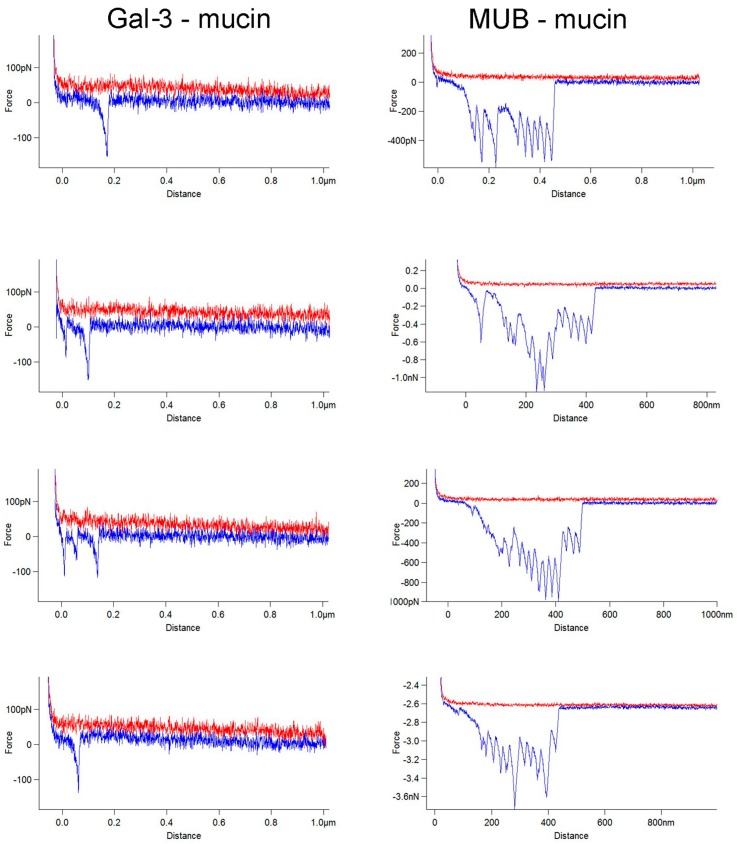
Comparison of force curves of the interactions between (**left**) Gal-3 and mucin and (**right**) MUB and mucin. These illustrate the differences between the molecular unfolding events of MUB and occasional, but non-symmetrical, multiple adhesive events of Gal-3 in each of their retraction force-distance curves as they interact with mucin.

In order to further support the validity of this model, we compared the binding properties of mucin to Gal-3, a mammalian lectin previously shown to bind to mucin [51,52] and characterised by the presence of one carbohydrate-binding domain (CRD) [53]. Gal-3 bound to immobilised pPGM in a fashion similar to the one reported with plant lectins [42] (Figure 7).

Figure 7 shows a typical set of force curves obtained in the interactions between Gal-3 and mucin (Figure 7) and between MUB and mucin (Figure 7). The Gal-3-mucin force distance data showed a few occasional multiple adhesive interactions but these are minor in terms of numbers compared to MUB, in line with Gal-3 globular fold and unique CRD. In addition, the negative peaks in the Gal-3-mucin retraction curves have relatively large separation distances (sometimes > 100 nm) and lower adhesive magnitude as compared to the MUB–mucin data set. Together, these data suggest that the binding of Gal-3 to mucin is unlikely to be due to the unfolding events regularly seen in the strong MUB–mucin interactions. In contrast, it is expected that the significantly less frequent multiple interaction events that occur will generally be due to the presence of multiple receptor sugars in mucins. This is in line with previous results showing that separation distances dominate when molecules with a single CRD show multiple interactions with a receptor [42]. Here, a modal value of 46 nm was calculated for Gal-3-mucin separation events, corresponding to 4.3 per 200 nm, whereas the energy data, corresponding to the integration of the adhesive peaks, was 3.08 aJ.

**Figure 8:**
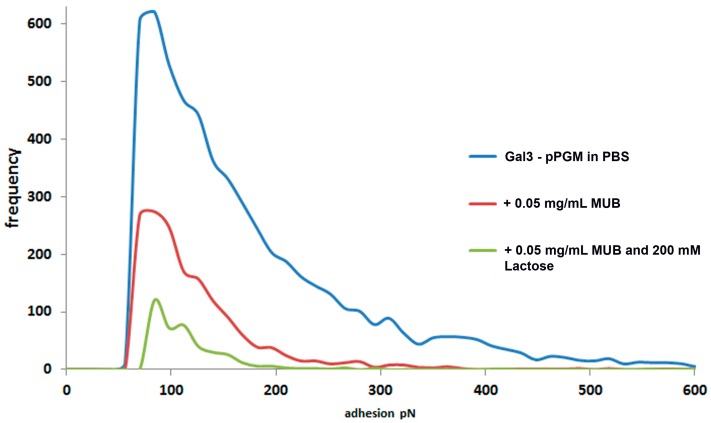
Interaction between Gal-3 and pPGM in the presence of MUB. Histogram quantifications illustrating the effects on the interaction between Gal-3 and pPGM following addition of free MUB and lactose into the solution.

To further explore the potential effect of MUB adhesive properties, MUB was added to the solution in the AFM liquid cell at a low concentration (0.05 mg·mL^−1^) during the interaction between an AFM tip functionalised with Gal-3 and a pPGM-coated slide. The quantification of the effect on adhesion is illustrated in Figure 8. Addition of MUB caused a significant reduction in the number of adhesive events (from 621 to 274) and also reduced the range of binding magnitude to only five adhesions with a value of ~364 pN. Addition of lactose, a known Gal-3 ligand, to the solution caused a further reduction in adhesion frequency and magnitude, confirming the validity of the affinity data.

## 3. Discussion

The MUB family of adhesins is characterized by the type and number of discrete modules, with large numbers of Mub repeats occurring in proteins from bacterial species inhabiting the GI tract, supporting their importance in mediating host–microbe interactions [26,54]. Our single-molecule experiments provide novel insights into the molecular mechanisms of MUB-mediated adhesion to mucins. Our main finding is that MUB exhibits remarkable adhesive properties occurring through sequential unfolding of the Mub repeats. Using force spectroscopy, we showed that many repetitive unfolding events occur during binding to mucins as shown in the negative peak quantification in the retraction force-distance curves collected at a scan rate of 1 Hz in the “force-volume” maps. We observed several consecutive unfolding events in the force-distance measurements during the “force-volume” mapping of the scanned mucin areas which may indicate that several MUB molecules may be present on the functionalised AFM tip. The measured forces were specific as they were essentially abolished in the presence of anti-Mub antibodies. Previous force spectroscopy studies showed that the number of negative peaks in the retraction force curves reveals how many of the domains are unfolded [48]. In addition, it was recently shown that a multi-domain polyprotein which is composed of two domains shows two sets of unfolding peaks which are discriminatory in terms of their size [55]. In that study, the authors suggested that the length is maintained by using intrinsic disorder to form highly cooperative and stable interfaces that mediate communication between non-adjacent stiff domains. This is consistent with the data in our study that show the relationship between the structure and function of this highly-active bacterial adhesin.

The extended conformation of MUB has been demonstrated by the resolution of the three dimensional structure of a type 1 repeat (PDB entry ID 4MT5) [26] and a type 2 repeat (PDB entry ID 3I57) [56], both forming an elongated structure of 110 Å in length and 24 Å in diameter. It is interesting to note that the two Mub types are very similar in terms of structure with a structural alignment Z-score of 15.1 over 176 aligned residues (rmsd 4.1 Å) while sharing 42% sequence identity [26]. It is tempted to speculate that the two sets of unfolding peaks observed upon retraction may correspond to the respective unfolding of Mub type 1 and Mub type 2 repeats, although this would require further investigations. The extended conformation of the full-length MUB was further supported by SAXS experiments of double and triple Mub repeats, demonstrating a tandem-repeat organization characteristic of extended rod-shaped proteins, and by AFM of the whole MUB molecule demonstrating a 'beads on a string' arrangement of repeats, generating 174 nm long protein fibrils [26]. The separation distance values of the unfolding peaks (24.3 ± 2.9 nm) are in reasonable agreement with the size of the 14 Mub repeats (each of about 11 nm in length as determined by X-ray crystallography and SAXS studies) [26] since they are extended by the unfolding. The sum of the distances that the multiple negative peaks cover suggests an average length of 284 ± 79 nm which indicates that the unfolding distance of the repeat domains has an average of 221%. The binding mechanism reported here may explain why commensal bacteria have evolved to express linear, multi-repeat structures which could match the multiple receptor positions along the mucin glycan chains, in contrast to pathogenic bacteria which typically possess lectins which are located at the N-terminal end of adhesins. The presence of multiple binding sites along the length of MUB protein may strengthen binding and confine the bacteria to the outer mucus layer. These data strongly support the binding model we previously proposed between *L. reuteri* ATCC 53608 MUB and mucin [26]. Such a mechanism is reminiscent of pili-mediated *Lactobacillus rhamnosus* GG (LGG) interactions with biotic (mucin, intestinal cells) or abiotic (hydrophobic monolayers) surfaces [13,57]. However, although MUB is similar to pili in terms of overall structural conformation (linear and modular), there are major differences in terms of the nature and biosynthesis of the domains constituting these adhesins [11,26] which are reflected by the force spectroscopy data. LGG pili are composed of three pilin subunits, SpaA/SpaB/SpaC with mucus adhesion properties conferred by the minor pilin SpaC (895 aa). Although a SpaC three-dimensional structure is not available, it is predicted to display a modular structure of tandem Ig-like domains joined end-to-end through flexible regions and stabilized by intramolecular isopeptide bonds, a feature of Gram-positive bacterial pili [11]. In line with this, at the single pilin level, AFM revealed that purified monomeric SpaC could not be unfolded, even by large mechanical forces [41], whereas our data showed sequential unfolding events. In addition, the binding forces between SpaC pilins (attached on AFM tips) and immobilised mucin showed a mean adhesion of 64 ± 20 pN (at 1000 nm/s) and a “rupture” length in the 10–200 nm range [41], which contrasts with the high strengths (380–482 pN) and “rupture” length (14–1019 nm) reported here for MUB full-length protein. It is of note that the retraction speed used in our study was double the rate (2000 nm/s) therefore generating larger adhesion force. However, since the “rupture” force of receptor-ligand interactions varies logarithmically with loading rates [58], a logarithmic increase of 0.7 (corresponding to the rate difference) would not account for the increase in “rupture” force from 64 to 380 pN if SpaC and MUB adhesion to mucin had a similar binding strength. MUB thus appears to bind mucin more strongly than SpaC. Together, these results can be explained by (i) the size and number of repeats involved in the MUB–mucin experiments (14-repeat domains of 183–206 amino acids in length), as compared with mucin adhesion to monomeric SpaC; and (ii) by the absence of flexible linker molecules between Mub repeats, as shown by SAXS analysis [26], impeding a potential elongation of the MUB molecule up to 212%. Indeed, pulling experiments on living bacteria showed that LGG displayed two mechanical properties: a zipper-like adhesion, reflecting the distribution of multiple SpaC molecules along the pilus length, and nanospring properties, reflecting the flexible nature of pili [41]. On hydrophobic surfaces, bacterial pili strengthened adhesion through remarkable nanospring properties, whereas on mucin, nanosprings were more frequent and adhesion forces larger, reflecting the influence of specific pili–mucin bonds [57]. In contrast, sequential unfolding of repeats from a large adhesin LapA (~520 kDa) from *Pseudomonas fluorescens* was only observed upon interaction of the bacterial cells with hydrophilic substrates and not with mucins, reflecting the contribution of *P. fluorescens* LapA to substrate attachment and biofilm formation [59]. Our results are in support of a nanospring adhesion model between MUB and mucin, mediated by unfolding of the multiple Mub repeats constituting the adhesin. This was further supported by force spectroscopy experiment of MUB–MUB interactions where the numbers of unfolding events in the force volume maps were 71% of the 1024 force curves. The combination of data in the measurements of MUB self-interactions provides further evidence in support of a multiple binding model of MUB.

This proposed mechanism was further explored by comparing the adhesion behaviour of a single CRD lectin, Gal-3, to pPGM under the same experimental conditions. We previously showed that pPGM was recognised by a range of plant lectins including *Ricinus communis* agglutinin I (RCA), peanut (*Arachis hypogaea*) agglutinin (PNA), *Maackia amurensis* lectin II (MALII), and *Ulex europaeus* agglutinin I (UEA) [42]. Binding energy of 4, 1.6, and 26 aJ was determined on pPGM for RCA, PNA, and UEA. The distributions of the nearest binding site separations estimated the number of binding sites in a 200-nm mucin segment to be 4 for RCA, PNA, and UEA, and 1.8 for MALII. Here we showed that Gal-3 followed a similar adhesion pattern with 4.3 separation events per 200 nm, which is in accordance with 46 nm and a binding energy of 0.79 aJ.

Our data provide evidence in support of the multiple binding model of MUB to terminal sugars present on the glycan antennae of the mucin chains [26]. Binding was decreased (but not abolished) by competition with free sialylated ligands, suggesting that higher concentrations of sugars may be required for inhibition or that MUB may recognize additional sugars on the mucin glycan chains. This multivalency is likely to explain the high strength of MUB binding to mucin reported here as compared to more common protein ligand-carbohydrate receptor interactions [60]. Presumably, this mechanism enables the bacteria to resist high shear forces within the GI tract environment, as previously suggested for pili-mediated adhesion of *L. rhamnosus* GG using non-invasive single-cell force spectroscopy [57].

Given that the composition of the mucus-associated microbiota is distinct to that of the lumen [61], it is tempting to postulate that mucin glycans could be an important contributing factor for this selection [62]. This is further supported by studies showing differences in *O*-glycosylation between the colon MUC2 of different species and a uniform glycosylation across human sigmoid colons [63]. Thus, it is hypothesized that bacterial adhesion to host mucin glycans is a selection mechanism mediated by specific mucus adhesins. The approach presented here provides a means to further address the mechanism for this selection using a range of mucins with different glycosignatures, such as those in IBD patients.

## 4. Materials and Methods

### 4.1. Materials

Porcine gastric mucin (PGM) type III, Dulbecco’s phosphate buffered saline (PBS), HEPES buffered saline, CaCl_2_, and MnCl_2_ were purchased from Sigma Aldrich (Dorset, UK). PGM was further purified (pPGM) as previously described [64]. α2,3- and α2,6-sialyllactose were purchased from Carbosynth (Berkshire, UK). Anti-Mub antibodies against Mub1 and Mub2 repeats were produced as previously reported [25]. Lactose was purchased from Fluka Chemicals Ltd. (Gillingham, UK).

### 4.2. Full-Length MUB Purification

MUB protein was purified from the spent culture of *L. reuteri* ATCC 53608 grown in Lactobacillus defined (LDMII) media as previously described [24]. Briefly, bacteria were grown 22–24 h stationary at 37 °C in an anaerobic cabinet. Cells were removed by centrifugation at 8000× *g*, 15 min and 4 °C, and the supernatant clarified by vacuum filtration on a 0.45 µm filter followed by a 0.22 µm filter (Sigma Aldrich). Initially, ammonium sulphate was added to 20% *w*/*v*, stirred at 4 °C for 1 h and centrifuged at 10 000× *g* for 15 min at 4 °C to remove the majority of proteins, lipids, and glycolipids not of interest. Protein precipitation was then performed by increasing the supernatant concentration to 60% *w*/*v* ammonium sulphate (Sigma Aldrich, Dorset, UK) and stirring overnight at 4 °C. Total protein was resuspended in 10 mL of 20% ammonium sulphate, to which 10 ml of *t*-butanol (Sigma Aldrich) was added and vortexed vigorously for 1 min. The solution was centrifuged at 3000× *g* for 5 min, resulting in a two-phase solution with the protein of interest recovered from the layer interface. The protein interface was resuspended in phosphate buffered saline (PBS) at pH 7.4 and concentrated to 1 mL using a Vivaspin 6 spin filter of 100 kDa cut-off (Sigma Aldrich). CHAPS [3-[(3-Cholamidopropyl)dimethylammonio]-1-propanesulfonate hydrate] was added to 0.5% (*w*/*v*) and the solution purified by size exclusion chromatography using a Superose 6 10/300 GL column (GE Healthcare Life Sciences, Little Chalfont, Buckinghamshire, UK). MUB protein elutes in the void volume and was confirmed by SDS-PAGE. MUB was dialysed extensively against PBS (pH 7.4) to remove CHAPS prior to experimental use.

### 4.3. Gal-3 Heterologous Expression and Purification 

The His-Gal-3 expression strain (Tuner DE3) was grown overnight in Luria-Bertani (LB) medium supplemented with 1% glucose and 100 µg/mL carbenicillin at 37 °C in a shaking incubator at 200 rpm. The culture was used to inoculate the medium at a 1/50 dilution and grown until reaching an OD600 of 1.0 (1.5–2 h). After addition of isopropyl β-d-1-thiogalactopyranoside (IPTG; Melford Laboratories, Ipswich, UK) (1 mM), induction of protein expression was allowed to proceed for 3–4 h at 37 °C while shaking at 200 rpm. Cells were collected after centrifugation at 8000× *g*, 4 °C and 15 min, and resuspended in a 20 mM Tris-HCl, 500 mM NaCl solution (pH 7.9) prior to cell lysis. Sonication was carried out on a Soniprep 150 (MSE (UK)) utilising 10 × 15 s intervals, each followed by 30 s on ice. Cell debris were removed by centrifugation and the supernatant applied to a nickel-nitrilotriacetic acid (Ni-NTA) affinity column pre-equilibrated in buffer (20 mM Tris-HCl, 500 mM NaCl, pH 7.9). The column was washed with 5 column vol. using the same buffer and the His-tagged protein eluted with an increased concentration of imidazole (20 mM Tris-HCl, 500 mM NaCl, 500 mM imidazole, pH 7.9). The protein was buffered exchanged to PBS and applied to a 2 mL lactose-agarose column (Sigma Aldrich). The column was then washed with 5 column vol. of PBS (pH 7.4) and the protein eluted in PBS containing 200 mM lactose. Protein purity was assessed by SDS-PAGE. The purified protein was buffer exchanged against PBS (pH 7.4) to remove lactose prior to experimental use.

### 4.4. Atomic Force Microscopy

The atomic force microscope used in this study was an MFP-3D BIO (Oxford Instruments Company Asylum Research, Santa Barbara, CA, USA). The interactions were examined by covalently attaching the purified MUB or Gal-3 molecules to AFM tips and the mucin molecules to glass slides to enable binding interactions to be measured in a specific manner [38]. An additional experiment was to covalently attach the same set of the purified MUB to both AFM tips and glass slides. Silicon nitride AFM tips (PNP-TR, Nanoworld AG, Neuchâtel, Switzerland) and freshly cleaned glass slides were functionalized using a four step procedure (carried out at 21 °C): the first step involved incubation in a 2% solution of 3-mercaptopropyltrimethoxy silane (MTS, Sigma-Aldrich, Poole, UK) in toluene (dried over a 4 Å molecular sieve) for 2 h, followed by washing with toluene and then chloroform. In the second step, the silanised tips were incubated for 1 h in a 1 mg·mL^−1^ solution of a heterobifunctional linker: MAL-PEG-SCM, 2 kD (Creative PEGWorks, NC, USA) in chloroform. The silanised glass slides were incubated in 5 mM *N*-γ-maleimidobutyryloxy-succinimide ester (GMBS) in ethanol (Thermo Fisher Scientific, Waltham, MA, USA). The tips were rinsed with chloroform and the slides with ethanol, and then dried with argon. The third step involved covalent attachment of the molecules investigated in this study by incubation of the tips/slides in 1 mg·mL^−1^ solutions of the proteins in PBS (pH 7.4) for 1 h at 21 °C, followed by a PBS washing step. The fourth step involved incubation of the functionalized cantilevers/slides in a 10 mg/mL solution of glycine in PBS to “amine”-cap any unreacted succinimide groups, followed by washing in PBS. Binding measurements were carried out in PBS (pH 7.4).

### 4.5. Force Spectroscopy

The experimental data were captured in “force-volume” (FV) mode at a rate of 2 μm·s^−1^ in the *Z* direction and at a scan rate of 1 Hz and a maximum load force of 300 pN (pixel density of 32 × 32 which collects 1024 force-distance curves). The spring constant, k, of the cantilevers was determined by fitting the thermal noise spectra [65], yielding typical values in the range 0.03–0.06 N·m^−1^. Adhesion in the force spectra was quantified using a bespoke Excel macro [66] which fits a line to the baseline of the retract portion of the force-distance data. The interaction force was calculated by fitting a straight line to the “off” region of the retract curve and then calculating the magnitude of specific adhesion peaks, which appeared after the AFM tip had broken contact with the glass surface. This approach ensured any non-specific tip-sample interactions (which appear at the tip-glass detachment-point) were eliminated from the measurements. The separation distances between individual adhesive events in the force spectra were also determined using the macro. Peak identification in the retract data was carried out by identifying turning points, and discrimination from noise was achieved through the use of a user-adjustable value, which set the threshold level in terms of a multiple of the amplitude of the noise level in the data (typically 6×). The macro peak identification enabled discrimination of the negative peak reflecting disconnection of the molecules on the AFM tip and glass slides. The last peak in each of the multiple adhesion events was quantified for “rupture” force between the interacting molecules. In addition the number of unfolding events was quantified by selecting force curves which had >5 multiple negative peaks with matching separation distances to discriminate symmetrical from non-symmetrical multiple adhesion events.

For competition experiments, the force measurements were repeated on the same region of the samples after addition of the free sugars (lactose 400 mM, 3’SL and 6’SL 200 mM), free MUB (0.1 mg·mL^−1^) or anti-Mub antibodies (0.1 mg·mL^−1^) to the AFM’s liquid cell, resulting in a final concentration of 200 mM lactose, 100 mM 3’SL and 6’SL, 0.05 mg·mL^−1^ MUB, 0.05 mg·mL^−1^ anti-Mub antibody. Following addition of the new compounds to the liquid cell a minimum incubation period of 15 min to allow sufficient mixing and settling of the AFM cantilever was included in the protocol.

## Supplementary Materials

Supplementary materials can be found at www.mdpi.com/1422-0067/17/11/1854/s1.

## References

[1] Bäckhed F., Ley R.E., Sonnenburg J.L., Peterson D.A., Gordon J.I. (2005). Host-bacterial mutualism in the human intestine. Science.

[2] Chang C., Lin H. (2016). Dysbiosis in gastrointestinal disorders. Best Pract. Res. Clin. Gastroenterol..

[3] Johansson M.E., Ambort D., Pelaseyed T., Schütte A., Gustafsson J.K., Ermund A., Subramani D.B., Holmén-Larsson J.M., Thomsson K.A., Bergström J.H. (2011). Composition and functional role of the mucus layers in the intestine. Cell. Mol. Life Sci..

[4] Brockhausen I., Schachter H., Stanley P., Varki A., Cummings R.D., Esko J.D. (2009). O-GalNAc glycans. Essentials of Glycobiology.

[5] Juge N. (2012). Microbial adhesins to gastrointestinal mucus. Trends Microbiol..

[6] Tailford L.E., Crost E.H., Kavanaugh D., Juge N. (2015). Mucin glycan foraging in the human gut microbiome. Front. Genet..

[7] Gerbino E., Carasi P., Mobili P., Serradell M.A., Gómez-Zavaglia A. (2015). Role of S-layer proteins in bacteria. World J. Microbiol. Biotechnol..

[8] Hymes J.P., Johnson B.R., Barrangou R., Klaenhammer T.R. (2016). Functional analysis of an S-Layer-associated fibronectin-binding protein in *Lactobacillus acidophilus* NCFM. Appl. Environ. Microbiol..

[9] Granato D., Perotti F., Masserey I., Rouvet M., Golliard M., Servin A., Brassart D. (1999). Cell Surface-Associated Lipoteichoic Acid Acts as an Adhesion Factor for Attachment of *Lactobacillus johnsonii* La1 to Human Enterocyte-Like Caco-2 Cells. Appl. Environ. Microbiol..

[10] Walter J., Loach D.M., Alqumber M., Rockel C., Hermann C., Pfitzenmaier M., Tannock G.W. (2007). d-alanyl ester depletion of teichoic acids in *Lactobacillus reuteri* 100–23 results in impaired colonization of the mouse gastrointestinal tract. Environ. Microbiol..

[11] Kankainen M., Paulin L., Tynkkynen S., von Ossowski I., Reunanen J., Partanen P., Satokari R., Vesterlund S., Hendrickx A.P., Lebeer S. (2009). Comparative genomic analysis of *Lactobacillus rhamnosus* GG reveals pili containing a human-mucus binding protein. Proc. Natl. Acad. Sci. USA.

[12] Von Ossowski I., Reunanen J., Satokari R., Vesterlund S., Kankainen M., Huhtinen H., Tynkkynen S., Salminen S., de Vos W.M., Palva A. (2010). Mucosal adhesion properties of the probiotic *Lactobacillus rhamnosus* GG SpaCBA and SpaFED pilin subunits. Appl. Environ. Microbiol..

[13] Castelain M., Duviau M.P., Oxaran V., Schmitz P., Cocaign-Bousquet M., Loubière P., Piard J.C., Mercier-Bonin M. (2016). Oligomerized backbone pilin helps piliated *Lactococcus lactis* to withstand shear flow. Biofouling.

[14] Nishiyama K., Ueno S., Sugiyama M., Yamamoto Y., Mukai T. (2016). *Lactobacillus rhamnosus* GG SpaC pilin subunit binds to the carbohydrate moieties of intestinal glycoconjugates. Anim. Sci. J..

[15] Suzuki K., Nishiyama K., Miyajima H., Osawa R., Yamamoto Y., Mukai T. (2016). Adhesion properties of a putative polymorphic fimbrial subunit protein from *Bifidobacterium longum* subsp. *longum*. Biosci. Microbiota Food Health.

[16] Granato D., Bergonzelli G.E., Pridmore R.D., Marvin L., Rouvet M., Corthésy-Theulaz I.E. (2004). Cell surface-associated elongation factor Tu mediates the attachment of *Lactobacillus johnsonii* NCC533 (La1) to human intestinal cells and mucins. Infect. Immun..

[17] Bergonzelli G.E., Granato D., Pridmore R.D., Marvin-Guy L.F., Donnicola D., Corthésy-Theulaz I.E. (2006). GroEL of *Lactobacillus johnsonii* La1 (NCC 533) is cell surface associated: potential role in interactions with the host and the gastric pathogen *Helicobacter pylori*. Infect. Immun..

[18] Kinoshita H., Uchida H., Kawai Y., Kawasaki T., Wakahara N., Matsuo H., Watanabe M., Kitazawa H., Ohnuma S., Miura K. (2008). Cell surface *Lactobacillus plantarum* LA 318 glyceraldehyde-3-phosphate dehydrogenase (GAPDH) adheres to human colonic mucin. J. Appl. Microbiol..

[19] Nishiyama K., Ochiai A., Tsubokawa D., Ishihara K., Yamamoto Y., Mukai T. (2013). Identification and characterization of sulfated carbohydrate-binding protein from *Lactobacillus reuteri*. PLoS ONE.

[20] Patel D.K., Shah K.R., Pappachan A., Gupta S., Singh D.D. (2016). Cloning, expression and characterization of a mucin-binding GAPDH from *Lactobacillus acidophilus*. Int. J. Biol. Macromol..

[21] Petrova M.I., Imholz N.C., Verhoeven T.L., Balzarini J., van Damme E.J., Schols D., Vanderleyden J., Lebeer S. (2016). Lectin-Like molecules of *Lactobacillus rhamnosus* GG inhibit pathogenic *Escherichia coli* and *Salmonella* biofilm formation. PLoS ONE.

[22] Valeriano V.D., Bagon B.B., Balolong M.P., Kang D.K. (2016). Carbohydrate-binding specificities of potential probiotic *Lactobacillus* strains in porcine jejunal (IPEC-J2) cells and porcine mucin. J. Microbiol..

[23] Etzold S., Juge N. (2014). Structural insights into bacterial recognition of intestinal mucins. Curr. Opin. Struct. Biol..

[24] Roos S., Jonsson H. (2002). A high-molecular-mass cell-surface protein from *Lactobacillus reuteri* 1063 adheres to mucus components. Microbiology.

[25] MacKenzie D.A., Jeffers F., Parker M.L., Vibert-Vallet A., Bongaerts R.J., Roos S., Walter J., Juge N. (2010). Strain-specific diversity of mucus-binding proteins in the adhesion and aggregation properties of *Lactobacillus reuteri*. Microbiology.

[26] Etzold S., Kober O.I., MacKenzie D.A., Tailford L.E., Gunning P., Walshaw J., Hemmings A.M., Juge N. (2014). Structural basis for adaptation of lactobacilli to gastrointestinal mucus. Environ. Microbiol..

[27] Lukić J., Strahinić I., Jovčić B., Filipić B., Topisirović L., Kojić M., Begović J. (2012). Different roles for lactococcal aggregation factor and mucin binding protein in adhesion to gastrointestinal mucosa. Appl. Environ. Microbiol..

[28] Kinoshita H., Uchida H., Kawai Y., Kitazawa H., Miura K., Shiiba K., Horii A., Saito T. (2007). Quantitative evaluation of adhesion of lactobacilli isolated from human intestinal tissues to human colonic mucin using surface plasmon resonance (BIACORE assay). J. Appl. Microbiol..

[29] Huang I.N., Okawara T., Watanabe M., Kawai Y., Kitazawa H., Ohnuma S., Shibata C., Horii A., Kimura K., Taketomo N. (2013). New screening methods for probiotics with adhesion properties to sialic acid and sulphate residues in human colonic mucin using the Biacore assay. J. Appl. Microbiol..

[30] Nishiyama K., Kawanabe A., Miyauchi H., Abe F., Tsubokawa D., Ishihara K., Yamamoto Y., Mukai T. (2014). Evaluation of bifidobacterial adhesion to acidic sugar chains of porcine colonic mucins. Biosci. Biotechnol. Biochem..

[31] Nishiyama K., Nakamata K., Ueno S., Terao A., Aryantini N.P., Sujaya I.N., Fukuda K., Urashima T., Yamamoto Y., Mukai T. (2015). Adhesion properties of *Lactobacillus rhamnosus* mucus-binding factor to mucin and extracellular matrix proteins. Biosci. Biotechnol. Biochem..

[32] Naughton J., Duggan G., Bourke B., Clyne M. (2014). Interaction of microbes with mucus and mucins: Recent developments. Gut Microbes.

[33] Jeffers F., Fuell C., Tailford L.E., MacKenzie D.A., Bongaerts R.J., Juge N. (2010). Mucin-lectin interactions assessed by flow cytometry. Carbohydr. Res..

[34] Naughton J.A., Mariño K., Dolan B., Reid C., Gough R., Gallagher M.E., Kilcoyne M., Gerlach J.Q., Joshi L., Rudd P. (2013). Divergent mechanisms of interaction of *Helicobacter pylori* and *Campylobacter jejuni* with mucus and mucins. Infect. Immun..

[35] Lukic J., Strahinic I., Milenkovi M., Nikolic M., Tolinacki M., Kojic M., Begovic J. (2014). Aggregation factor as an inhibitor of bacterial binding to gut mucosa. Microb. Ecol..

[36] Altamimi M., Abdelhay O., Rastall R.A. (2016). Effect of oligosaccharides on the adhesion of gut bacteria to human HT-29 cells. Anaerobe.

[37] Beaussart A., El-Kirat-Chatel S., Sullan R.M., Alsteens D., Herman P., Derclaye S., Dufrêne Y.F. (2014). Quantifying the forces guiding microbial cell adhesion using single-cell force spectroscopy. Nat. Protoc..

[38] Hinterdorfer P., Gruber H.J., Kienberger F., Kada G., Riener C., Broken C., Schindler H. (2002). Surface attachment of ligands and receptors for molecular recognition force microscopy. Colloids Surf..

[39] Dague E., Le D.T., Zanna S., Marcus P., Loubière P., Mercier-Bonin M. (2010). Probing in vitro interactions between *Lactococcus lactis* and mucins using AFM. Langmuir.

[40] Le D.T., Tran T.L., Duviau M.P., Meyrand M., Guérardel Y., Castelain M., Loubière P., Chapot-Chartier M.P., Dague E., Mercier-Bonin M. (2013). Unraveling the role of surface mucus-binding protein and pili in muco-adhesion of *Lactococcus lactis*. PLoS ONE.

[41] Tripathi P., Beaussart A., Alsteens D., Dupres V., Claes I., von Ossowski I., de Vos W.M., Palva A., Lebeer S., Vanderleyden J. (2013). Adhesion and nanomechanics of pili from the probiotic *Lactobacillus rhamnosus* GG. ACS Nano.

[42] Gunning A.P., Kirby A.R., Fuell C., Pin C., Tailford L.E., Juge N. (2013). Mining the “glycocode”—Exploring the spatial distribution of glycans in gastrointestinal mucin using force spectroscopy. FASEB J..

[43] Bhatia S.K., Shriver-Lake L.C., Prior K.J., Georger J.H., Calvert J.M., Bredehorst R., Ligler F.S. (1989). Use of thiol terminated silanes and heterobifunctional crosslinkers for immobilisation of antibodies on silica surfaces. Anal. Biochem..

[44] Kratky O., Porod G. (1949). Röntgenuntersuchung gelöster Fadenmoleküle. Recl. Trav. Chim..

[45] Flory P.J., Volkenstein M. (1969). Statistical mechanics of chain molecules. Biopolymers.

[46] Beuche F. (1962). Physical Properties of Polymers.

[47] Smith S.B., Cui Y., Bustamante C. (1996). Overstretching B-DNA: The elastic response of individual double-stranded and single-stranded DNA molecules. Science.

[48] Rounsevell R., Forman J.R., Clarke J. (2004). Atomic Force Microscopy: Mechanical unfolding of proteins. Methods.

[49] Morris V.J., Kirby A.R., Gunning A.P. (2009). Atomic Force Microscopy for Biologists.

[50] Gunning A.P., Pin C., Morris V.J. (2013). Galectin 3-β-galactobiose interactions. Carbohydr. Polym..

[51] Bresalier R.S., Byrd J.C., Wang L., Raz A. (1996). Colon cancer mucin: A new ligand for the β-galactoside-binding protein galectin-3. Cancer Res..

[52] Shan M., Gentile M., Yeiser J.R., Walland A.C., Bornstein V.U., Chen K., He B., Cassis L., Bigas A., Cols M. (2013). Mucus enhances gut homeostasis and oral tolerance by delivering immunoregulatory signals. Science.

[53] Dumic J., Dabelic S., Flögel M. (2006). Galectin-3: An open-ended story. Biochim. Biophys. Acta.

[54] Boekhorst J., Helmer Q., Kleerebezem M., Siezen R.J. (2006). Comparative analysis of proteins with a mucus-binding domain found exclusively in lactic acid bacteria. Microbiology.

[55] Gruszka D.T., Whelan F., Farrance O.E., Fung H.K.H., Paci E., Jeffries C.M., Svergun D.I., Baldock C., Baumann C.G., Brockwell D.J. (2015). Cooperative folding of intrinsically disordered domains drives assembly of a strong elongated protein. Nat. Commun..

[56] MacKenzie D.A., Tailford L.E., Hemmings A.M., Juge N. (2009). Crystal structure of a mucus-binding protein repeat reveals an unexpected functional immunoglobulin binding activity. J. Biol. Chem..

[57] Sullan R.M., Beaussart A., Tripathi P., Derclaye S., El-Kirat-Chatel S., Li J.K., Schneider Y.J., Vanderleyden J., Lebeer S., Dufrêne Y.F. (2014). Single-cell force spectroscopy of pili-mediated adhesion. Nanoscale.

[58] Strunz T., Oroszlan K., Schumakovitch I., Güntherodt H.J., Hegner M. (2000). Model energy landscapes and the force-induced dissociation of ligand-receptor bonds. Biophys. J..

[59] El-Kirat-Chatel S., Beaussart A., Boyd C.D., O'Toole G.A., Dufrêne Y.F. (2014). Single-cell and single-molecule analysis deciphers the localization, adhesion, and mechanics of the biofilm adhesin LapA. ACS Chem. Biol..

[60] Touhami A., Hoffmann B., Vasella A., Denis F., Dufrene Y.F. (2003). Probing specific lectin-carbohydrate interactions using atomic force microscopy imaging and force measurements. Langmuir.

[61] Li H., Limenitakis J.P., Fuhrer T., Geuking M.B., Lawson M.A., Wyss M., Brugiroux S., Keller I., Macpherson J.A., Rupp S. (2015). The outer mucus layer hosts a distinct intestinal microbial niche. Nat. Commun..

[62] Johansson M.E., Phillipson M., Petersson J., Velcich A., Holm L., Hansson G.C. (2008). The inner of the two Muc2 mucin-dependent mucus layers in colon is devoid of bacteria. Proc. Natl. Acad. Sci. USA.

[63] Larsson J.M., Karlsson H., Sjövall H., Hansson G.C. (2009). A complex, but uniform *O*-glycosylation of the human MUC2 mucin from colonic biopsies analyzed by nanoLC/MSn. Glycobiology.

[64] Crost E.H., Tailford L.E., Le Gall G., Fons M., Henrissat B., Juge N. (2013). Utilisation of mucin glycans by the human gut symbiont *Ruminococcus gnavus* is strain-dependent. PLoS ONE.

[65] Hutter J.L., Bechhoefer J. (1993). Calibration of atomic force microscope tips. Rev. Sci. Instrum..

[66] Gunning A.P., Chambers S., Pin C., Man A.L., Morris V.J., Nicoletti C. (2008). Mapping specific adhesive interactions on living human intestinal epithelial cells with atomic force microscopy. FASEB J..

