# Supplementary material for: Use of Atomic Force Microscopy to Study the Multi-Modular Interaction of Bacterial Adhesins to Mucins
Source: Int J Mol Sci. 2016 Nov 8;17(11):1854. doi: 10.3390/ijms17111854 (PMC5133854; doi:10.3390/ijms17111854)
Supplement: Supplementary file 1 [file ijms-17-01854-s001.pdf]

# Supplementary Materials: Use of Atomic Force Microscopy to Study the Multi-Modular Interaction of Bacterial Adhesins to Mucins

**A. Patrick Gunning, Devon Kavanaugh, Elizabeth Thursby, Sabrina Etzold, Donald A. MacKenzie and Nathalie Juge**

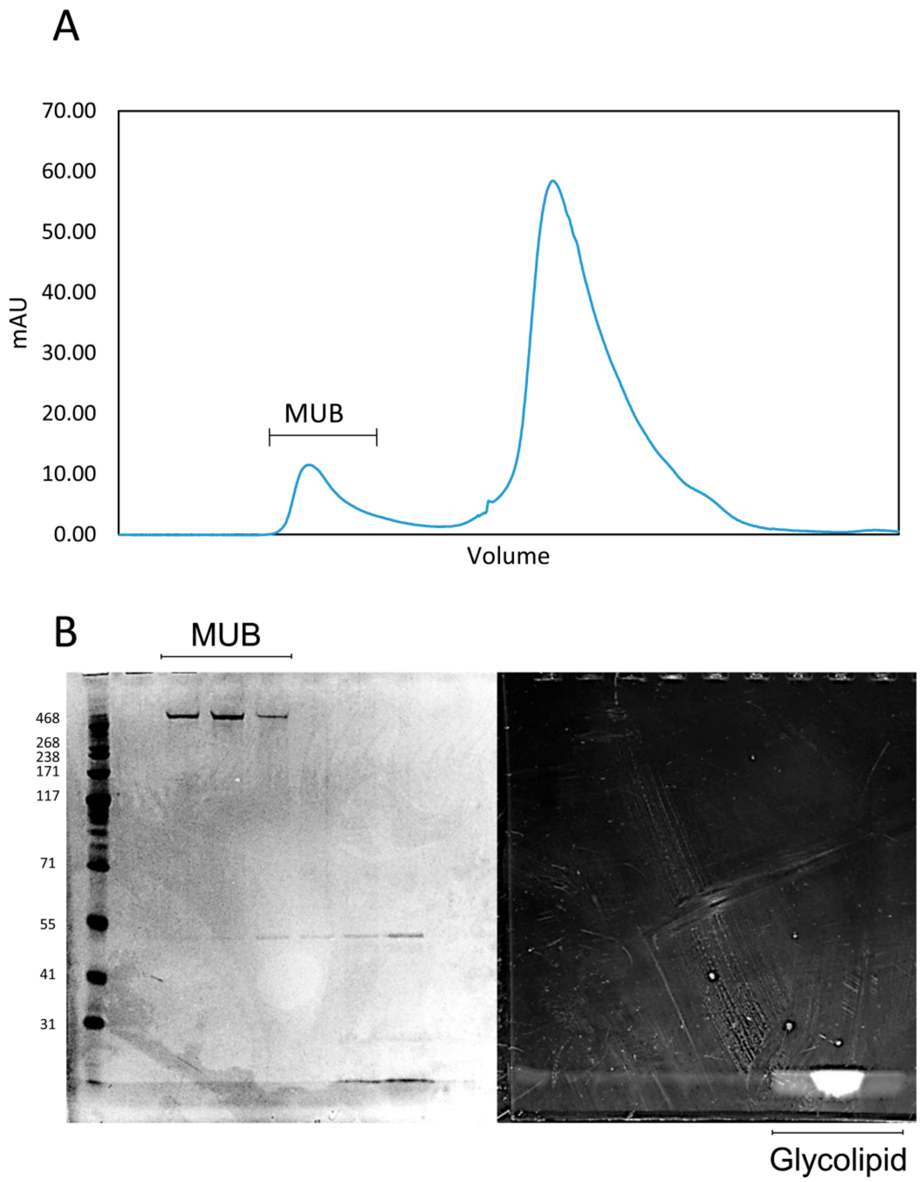


**Figure S1.** Elution and purification profiles of MUB. MUB is eluted in the void volume of the size exclusion chromatography column while the contaminating glycolipid emerges later in the chromatogram (**A**); SDS-PAGE confirms the purity of MUB: Coomassie-stained (**left**) and the effective separation from the glycolipid which forms a white precipitate in the gel front upon extended exposure to the staining solution (visualisation on clear plate (**right**)) (**B**).

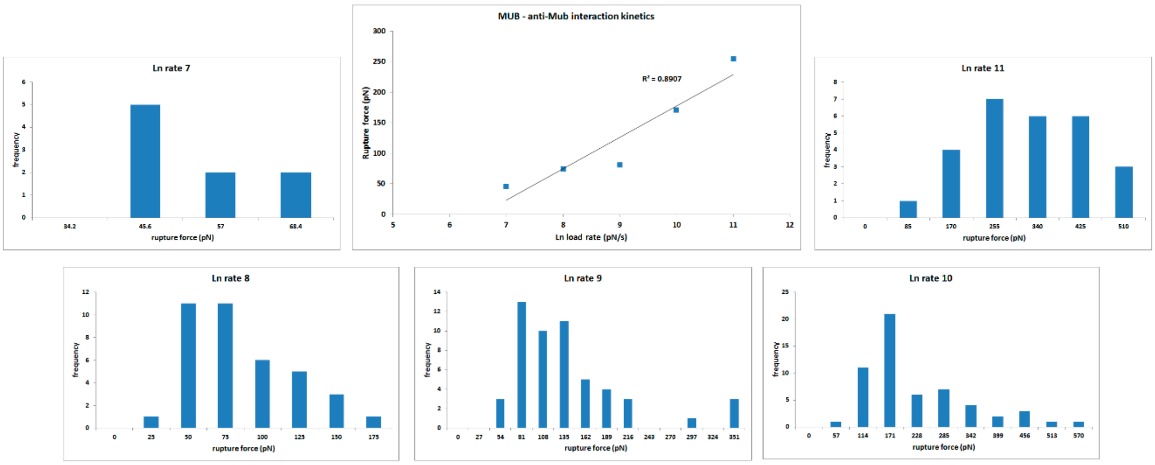


**Figure S2.** Analysis of the dissociation rates between MUB and anti-Mub antibody. The top centre panel is the main graph of rupture force (f) versus the logarithm of the loading rate ln(r) for the MUB—Anti-Mub antibody interactions which enables the calculation of interaction timescales.

The surrounding panels are histograms of quantification of the rupture force of the adhesion events in the gathered force distance curves at the five different retraction loading rates (df/dt) that were performed. The modal value of each set provides the data source for the main graph.
